# Utilization of Macroalgae for the Production of Bioactive Compounds and Bioprocesses Using Microbial Biotechnology

**DOI:** 10.3390/microorganisms11061499

**Published:** 2023-06-05

**Authors:** Seiji Shibasaki, Mitsuyoshi Ueda

**Affiliations:** 1 Laboratory of Natural Science, Faculty of Economics, Toyo University, Hakusan Bunkyo-ku, Tokyo 112-8606, Japan; 2 Office of Society-Academia Collaboration for Innovation (SACI), Kyoto University, Yoshidahonmachi, Sakyo-ku, Kyoto 606-8501, Japan; ueda.mitsuyoshi.7w@kyoto-u.ac.jp

**Keywords:** macroalgae, phlorotannin, molecular display, bioethanol, xylan, mannitol, laminarin, alginate

## Abstract

To achieve sustainable development, alternative resources should replace conventional resources such as fossil fuels. In marine ecosystems, many macroalgae grow faster than terrestrial plants. Macroalgae are roughly classified as green, red, or brown algae based on their photosynthetic pigments. Brown algae are considered to be a source of physiologically active substances such as polyphenols. Furthermore, some macroalgae can capture approximately 10 times more carbon dioxide from the atmosphere than terrestrial plants. Therefore, they have immense potential for use in the environment. Recently, macroalgae have emerged as a biomass feedstock for bioethanol production owing to their low lignin content and applicability to biorefinery processes. Herein, we provided an overview of the bioconversion of macroalgae into bioactive substances and biofuels using microbial biotechnology, including engineered yeast designed using molecular display technology.

## 1. Introduction

For many centuries, fossil fuel consumption has increased, leading to a high level of emissions of carbon dioxide into the atmosphere [1,2]. Moreover, human life relies on various materials produced via chemical synthesis using large amounts of energy. Recently, there has been an increase in energy demand in response to the growing global population and economy. Therefore, to pave the path to a sustainable future, it is critical we develop renewable and clean sources of bioenergy.

Bioethanol production has been proposed and developed using various agricultural biomasses, including corn [3], sugarcane [4], sugar beet [5], potato [6], and wheat [7]. Compared with fossil fuels, bioethanol produces fewer toxic substances and causes fewer harmful environmental issues [8]. However, concerns remain that the use of biomass for energy production competes with the use of food by humans and livestock. On the other hand, many biomolecules in macroalgae, including polysaccharides, can be converted to ethanol-fermentable sugars in ocean ecosystems. Therefore, macroalgae have attracted the attention of researchers as an alternative fuel source for bioethanol production. Macroalgae can grow at rates higher than those of terrestrial plants [9,10], and arable land is not needed for the cultivation or fertilization of macroalgae. Furthermore, macroalgae can grow in salt water, preventing competition for fresh water required for crop production in fields. Therefore, macroalgae are considered ideal resources for third-generation biofuels [11].

Algae are a group of photosynthetic, prokaryotic, and eukaryotic organisms [12]. Macroalgae come in different sizes and colors. They are classified according to their photosynthetic pigments, color schemes (red, brown, green, etc.), and habitat [9,13]. For example, the exclusive economic zone of Japan is approximately 450 million km^2^ (1/80 of the world’s total sea area), with >1000 macroalgal species in these limited areas. Furthermore, they have different chemical compositions and bioactive molecular contents.

**Figure 1:**
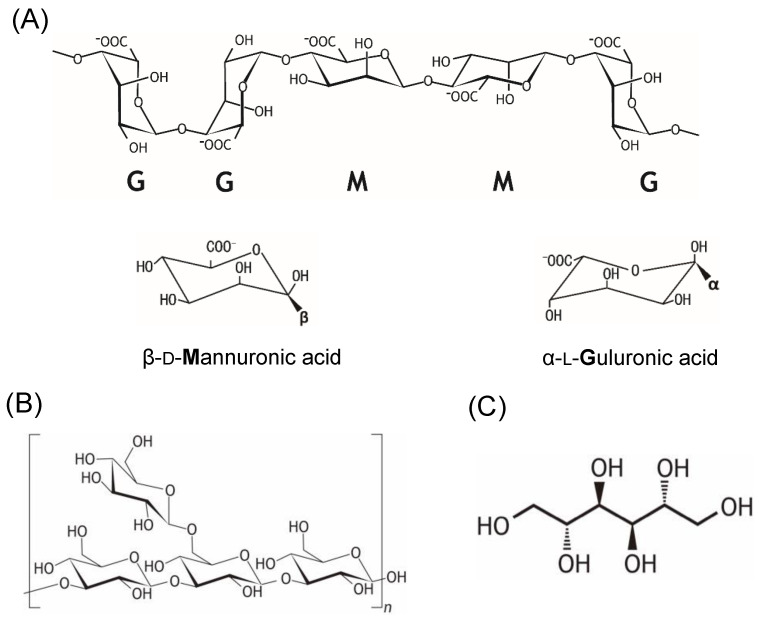
Examples of chemical structures of the saccharides in macroalgae. (**A**) Chain form (upper) and monomeric unit (lower) of alginate. (**B**) Laminarin. (**C**) Mannitol.

Polysaccharides represent biomass or bioactive compounds. Brown algae commonly contain laminarin and fucoidan, green algae contain ulvans, and red algae contain large amounts of carrageenan [14]. Brown macroalgae additionally contain alginate, cellulose, hemicellulose, laminarin, and mannitol, which are the major carbohydrates (Figure 1) and are characterized by high contents of mannitol, laminarin, and alginate [15]. Laminarin comprises a β-1,3-linked glucose polymer with connecting β-1,6 cross-linked branches. In brown macroalgae, it functions as a long-term storage compound and exhibits seasonal variations ranging from 0 to 35% on a dry basis [16]. Brown macroalgae additionally contain mannitol as a carbon storage compound, accounting for up to 20–30% of the dry weight [17].

Bioactive compounds such as proteins and peptides in macroalgae exhibit anti-inflammatory, antioxidant, antitumor, antiviral, neuroprotective, hypocholesterolemic, hepatoprotective, and liver-protecting functions [18]. These beneficial health effects are also mediated by specific diterpenes, pigments (fucoxanthin, phycocyanin, and carotenoids), polysaccharides, and bioactive peptides [19]. In particular, phenolic compounds have the most structural variation and highest content in macroalgae. Phlorotannins are the most widely investigated polyphenols, with high contents in brown macroalgae [20].

In the present review, we describe bioactive compounds and biofuels from macroalgae using microbial technology. Furthermore, we emphasize the applications of recent microbial biotechnologies and molecular display technologies for biofuel production from macroalgae.

## 2. Biological Activity and Bioconversion of Green Macroalgae

### 2.1. Ulvan

The green macroalgae *Ulva* species are edible seaweeds comprising health-promoting and bioactive compounds. The major carbohydrates of *Ulva* species are ulvans and glucans, with median values of 45.0 mol% and 22.5 mol% for rhamnose and glucuronic acid, respectively. Ulvan accounts for 9–36% of the dry weight of *Ulva* species [21]. It is high in dietary fiber, thereby promoting gastrointestinal health, and is associated with a decrease in the occurrence of chronic diseases. Dutschei et al. reported that *Bacillus licheniformis* can grow on a medium containing ulvan-derived xylose-containing oligosaccharides [22]. Heterologous expression of two marine enzymes, namely, ulvan lyase PL28 and glucuronyl hydrolase GH105 [23], in *Bacillus licheniformis* resulted in the efficient conversion of the algal polysaccharide ulvan as a carbon and energy source [22]. In another study on the saccharification of ulvans, a broad-spectrum ulvan lyase was identified (Cdf7993 protein) from *Formosa agariphila* KMM 3901 and investigated further [24].

### 2.2. L-Rhamnose

Rhamnose is an important monosaccharide that is widely distributed among microorganisms and plants. Certain bacterial saponin glycans contain rhamnolipids, mycolic acids, and extracellular polysaccharides [25]. In the green macroalga *Ulva lactuca*, L-rhamnose and D-glucose are the major carbohydrates present in the ulvan polysaccharide structure; these sugars can be recovered under mild conditions [26].

Investigation of the antiviral activity of rhamnose polysaccharides revealed that rhamnose sulfate in the green alga *Monostroma nitidum* exhibits anti-severe acute respiratory syndrome coronavirus 2 (SARS-CoV-2) activity. SARS-CoV-2 invasion is achieved via the interaction of its S-protein with angiotensin-converting enzyme 2 (ACE2) in susceptible host cells. The rhamnose fraction not only inhibited the binding of S-protein and ACE2 analogs but also that of SARS-CoV-2 and ACE2 analogs [27]. In addition, branched and sulfated heterorhamnan exhibited specific activity against the herpes simplex virus [28]. Sulfated polysaccharides, including L-rhamnose derived from the green alga *Spirogyra neglecta*, exhibit immunomodulatory activity [29]. In addition to the immunomodulatory activity, several green algae exert antitumor activity [30]. A study reported that extracts containing sulfated heterorhamnans from the green alga *Gayralia oxysperma* exerted cytotoxic effects against U-87 MG, a cell line isolated from a patient with malignant gliomas. Furthermore, sulfated polysaccharides with rhamnose increased the number of cells in the G1 phase [31].

*Clostridium beijerinckii* can use L-rhamnose as the sole carbon source to produce acetic acid, butyric acid [32], 1,2-propanediol, propionic acid, and n-propanol [33]. Green macroalgae can be processed into hydrolysates containing D-glucose and L-rhamnose; therefore, they have potential applications as an industrial fermentation strain. D-galactosyl-β1→4-rhamnose, which exerts immunomodulatory activity, is produced by a one-pot reaction using a combination of recombinant phosphorylases and dried baker’s yeast [34].

### 2.3. Bioconversion Using Yeast Cells

As an example of a yeast-based bioconversion application, Greetham et al. investigated the fermentation ability of the marine yeast *Wickerhamomyces anomalus* M15, particularly for hydrolysis and ethanol production, using brown (*Laminaria digitata*), green (*Ulva linza*), and red (*Porphyra umbilicalis*) macroalgae [35]. After pretreatment with seawater, the highest amount of sugar was liberated by the green macroalga *U. linza*. In addition, fermentation of *Wickerhamomyces anomalus* M15 using a concentrated hydrolysate from *Ulva linza* resulted in the production of 48.2 g/L ethanol, which is equivalent to an overall yield of 0.329 g/g available sugars [35]. As another yeast-based application, Jiang et al. investigated ethanol fermentation using a hydrolysate from the green macroalgae *Ulva*. They suggested that *Saccharomyces cerevisiae* RN1016 with xylose isomerase achieved the highest ethanol production levels among the microorganisms examined under the optimized thermochemical conditions [36].

## 3. Component and Bioconversion of Red Macroalgae

Carrageenan is the main carbohydrate component in red macroalgae such as *Eucheuma denticulatum* [37], and agar is the main component in species such as *Gelidium amansii* [38]. During the decomposition of agarose, enzymatic hydrolysis, acid hydrolysis, and acid prehydrolysis with subsequent enzymatic hydrolysis result in the liberation of 3,6-anhydro-α-L-galactose (AHG) and D-galactose for subsequent fermentation [39]. However, the decomposition of carrageenan is difficult because acid treatment produces inhibitory compounds such as acetic acid, furfural, 5-HMF, and levulinic acid [40,41,42]. Therefore, D-galactose and AHG from agarose are suitable target molecules in the use of red macroalgae.

Many marine microorganisms, including *Pseudoalteromonas carrageenovora* [43], *Zobellia galactanivorans* [44], *Pseudoalteromonas fuliginea* [45], and *Saccharophagus degradans* [46], exhibit agarase activity. Additionally, the catabolic pathway of AHG has been well investigated in the agarolytic marine bacteria *Vibrio* sp. [47] and *Streptomyces* sp. [48]. As an example of its application in bioconversion, the AHG catabolic pathway was introduced into an ethanologenic *Escherichia coli* strain. The engineered strain exhibited 2.0-fold higher AHG consumption and 1.2-fold higher ethanol production compared to the control [49].

As a yeast-based application in bioconversion, a study investigated ethanol production using a hydrolysate derived from the red macroalga *Gracilaria verrucosa* [50]. Analysis of the relationship between galactose adaptation effects and mRNA transcriptional levels revealed that the use of galactose for ethanol fermentation using *Gracilaria verrucosa* hydrolysates enhanced the overall ethanol yield in *Saccharomyces cerevisiae* KCCM 1129 [50]. In another bioconversion method using yeast cells, the representative probiotic yeast *S. cerevisiae* var. *boulardii* was used for depolymerization into a beneficial compound, neoagarooligosaccharides, by an endo-type β-agarase [51]. 

## 4. Bioactivity of Brown Macroalgae

### 4.1. Macroalgae Polyphenols or Phlorotannins

Polyphenols are compounds that contain more than two hydroxyl groups. Flavonoids, lignin, and tannins are well-known polyphenols produced by terrestrial organisms [52]. Tannins are further categorized into condensed tannins, which are formed by polymerized flavanols, and hydrolyzable tannins, which are combined with sugar and gallic or ellagic acid via ester bonds [53]. Polyphenols in macroalgae, known as phlorotannins, have a polymerized structure of phloroglucinol and are different from the tannins in terrestrial organisms. Eckols, phlorethols, fucols, fucophlorethols, fuhalols, isofuhalols, and carmalols are the basic structures of phlorotannins bound to phloroglucinol [54,55]. Physiological studies previously carried out on phlorotannins are described below.

### 4.2. Inhibiting Advanced Glycation End Product (AGE) Formation

AGEs are produced by nonenzymatic reactions between proteins and reducing sugars [56,57]. AGEs play important roles in the development of diabetic complications, osteoporosis, atherosclerosis, sarcopenia, and neuropathy [58,59]. Chemical synthesis has been used to develop glycation inhibitors to suppress AGE production. For example, aminoguanodine [60] and OPB-9195 [61] have been identified as AGE inhibitors; however, they have not been approved for clinical use owing to their adverse effects. Therefore, compounds that are effective against AGE formation have been explored in edible plants [62]. To this end, the antiglycation activities of phlorotannins present in brown macroalgae (*Ecklonia cava*, *Ecklonia kurome*, *Ecklonia stolonifera*, *Eisenia arborea*, and *Eisenia bicyclis*) have been studied.

### 4.3. Effect of Phlorotannins on Methylglyoxal (MGO) Formation

AGEs are produced after the formation of MGO, an α-dicarbonyl compound [63]. Studies have reported that the blood MGO levels were higher in patients with type I diabetes than in healthy people [64,65]. Therefore, phlorotannins extracted from Lessoniaceae were evaluated for their inhibitory activities against fluorescent AGE production in human and bovine serum albumin (HSA and BSA)–MGO models [66]. The inhibitory effect on the formation of fluorescent AGEs was calculated as the half-maximal inhibitory concentration (IC_50_). Compared with the positive control aminoguanidine hydrochloride (AG) (IC_50_ = 0.70 mg/mL in the HSA–MGO models and 0.9 mg/mL in the BSA–MGO models), phlorotannins from Lessoniaceae exhibited higher antiglycation activity (Table 1).

### 4.4. Effect of Phlorotannins on Glyceraldehyde (GA) Formation

GA is also involved in AGE production. AGEs derived from GA form faster than those from MGO [67]. Therefore, many studies have explored inhibitors of the formation of AGEs from GA [68,69,70]. In addition to the serum albumin–MGO models described in the previous section, the inhibitory effects of phlorotannins have been examined using HSA– or BSA–GA models [71]. As a result, phlorotannins from Lessoniaceae exhibited an IC_50_ of 0.48–0.70 mg/mL (Table 2). The inhibitory effect of phlorotannins derived from *Eisenia bicyclis* on fluorescent AGEs was 2.3–3.7-fold higher than that of AG as a positive control.

### 4.5. Effect of Phlorotannins on Nε-(Carboxymethyl)lysine (CML)

CML is an AGE formed by the oxidation of glucose with lysine [72]. In human dermal fibroblasts, CML–collagen decreased the ability of epidermal keratinocytes to adhere to collagen and induce apoptosis [73]. CML–collagen inhibits collagen cross-linking in osteoblasts and causes diabetic osteopenia [74,75]. The suppression of CML formation in these diseases is thought to be clinically important. Recently, the inhibitory effect of phlorotannins on CML formation was examined [76]. The inhibitory effect following treatment with phlorotannins from Lessoniaceae on CML formation was 0.16 μg/mL, which was distinctively lower than that following treatment with 0.40 mM AG as a positive control. Phloroglucinol and eckols inhibit CML formation at concentrations approximately 317–1818-fold lower than those of AG [76]. Taken together, phlorotannins can be considered potential inhibitors of CML formation.

## 5. Microbial Conversion of Macroalgae

### 5.1. Microorganisms and Their Enzymes

To develop bioconversion methods for algae, an effective method for crushing and saccharifying seaweed bodies is crucial. Considering these situations, algae-degrading microorganisms can be exploited to develop a sustainable tool for algal processing. Previous outbreaks of seaweed diseases have led to the screening of algae-degrading bacteria. The marine bacterium *Alteromonas elyakovii* KMM 162T was isolated from spot-wounded fronds of the brown macroalga *Laminaria japonica* [77]. Similar to other brown algae, *Fucus evanescens*-degrading bacteria, *Pseudoalteromonas* sp., and *Halomonas* sp. have also been isolated previously [78].

Tanaka et al. isolated four brown algae-degrading Gram-negative bacteria, namely *Formosa haliotis* strains from the gut of the abalone *Haliotis gigantea* [79]. Furthermore, they performed genomic analysis of the *Formosa haliotis* strain MA1 (LMG 28520T) to reveal the mechanism of degradation of seaweed bodies. As a result, more genes related to macromolecule degradation were identified compared with conventional marine bacteria [79]. Several genes related to hydrocarbon degradation and gene clusters related to alginate degradation have been identified.

Furthermore, genes encoding alginate lyase family PL-7, an oligoalginate lyase classified as alginate lyase (family PL-17), 4-deoxy-l-erythro-5-hexoseulose uronic acid (DEH) reductase, KdgF, 2-keto-3-deoxy-D-gluconate (KDG) kinase, and 2-dehydro-3-deoxy-phosphogluconate aldolase have been identified [80]. The KDG produced by this cluster is further metabolized in a major biochemical pathway of sugars. Using this gene cluster, *Formosa haliotis* may effectively and functionally use fewer compounds in marine environments than in terrestrial environments [80].

### 5.2. Degradation of Alginate

Alginate-degrading bacteria are considered industrially important because products using alginate lyases can be applied in the pharmaceutical industry, the food industry, and bioethanol production [81]. Several researchers have investigated alginolytic strains in the environment and identified them as *Sphingomonas* sp. strain A1 [82], *Zobellia galactanivorans* [83], *Vibrio splendidus* strain 12B01 [84], and *Saccharophagus degradans* strain 2–40 [85]. Alginate-degrading bacteria have been further explored for the efficient production of rare sugars from brown macroalgae by screening algae-corrupting bacteria. As a result of this screening, *Falsirhodobacter* sp. strain alg1 was isolated and analyzed [81,86]. Although there are only two alginate lyases, namely, AlyFRA and AlyFRB, in strain alg1, DEH was effectively produced by controlling the ratio of the two enzymes [81].

### 5.3. Immobilization of Recombinant Alginate Lyase

To achieve effective and sustainable DEH production, microbial strains of *Escherichia coli*, *Saccharomyces cerevisiae*, and *Sphingomonas* sp. A1 were developed by introducing genes encoding alginate lyase and other enzymes related to DEH fermentation and bioethanol production [87,88]. These strains can produce ethanol directly from sodium alginate. The enzymatic reactions of recombinant endo-alginate lyase Alg7D and exo-alginate lyase Alg17C from *Saccharophagus degradans* yielded 45.5% DEH (DEH weight/alginate weight) from alginate [89].

Considering the industrial applications of DEH, increasing the yield of DEH and examining the reusability of enzymes are warranted to minimize costs. In general, enzyme reusability can be attained by immobilizing the enzymes into carrier materials. Moreover, the immobilized enzymes can be handled as solids and readily separated from the reaction mixture containing the products. Tanaka et al. examined DEH production using free and immobilized alginate lyases, endo-type AlyFRA, and exo-type AlyFRB from *Falsirhodobacter* sp. alg1 [90].

The investigation using LC-MS revealed that the reaction of both recombinant enzymes rAlyFRA and rAlyFRB with sodium alginate generated highly purified DEH. Next, AlyFRA- and AlyFRB-immobilized enzymes with κ-carrageenan were prepared as iAlyFRA and iAlyFRB, respectively. The immobilization rates of AlyFRA and AlyFRB increased as the concentration of κ-carrageenan increased, and their κ-carrageenan gels were less fragile. Immobilized enzymes prepared with 4.0% (*w*/*v*) κ-carrageenan completely degraded the substrate and produced DEH in the seventh batch reaction [90]. Considering these facts, the immobilization of AlyFRA and AlyFRB can effectively and economically produce large amounts of DEH from sodium alginate. Therefore, the industrial-scale production of DEH via the extraction of saccharified liquid containing alginate from brown algae can be developed by improving the immobilization conditions and carrier materials.

## 6. Molecular Display Technology for Macroalgae Utilization

### 6.1. Technology for Immobilizing Proteins on the Cell Surface

After the development of genetic engineering, molecular display technology or cell surface engineering was developed for various biological investigations and was conveniently applied to prepare recombinant proteins in bioprocesses [91,92,93]. The first technology in this field was the so-called “phage display” technology, which was developed by Smith [94]. In this technology, a foreign protein is inserted into the filamentous phage protein III via genetic manipulation, and its fusion protein is produced on the virion surface. This technology is currently used for screening combinatorial proteins or clones of peptide ligands [95,96]; however, it is difficult to perform and involves steps such as infection of *Escherichia coli* cells with phages for the recovery of positive clones. This technical challenge can be solved using molecular display technology with bacterial cells, which can provide an easier display system without infection and can display large numbers of proteins [97,98].

Since the development of *E. coli* as a host bacterium for molecular display technology [99,100], several bacteria, such as *Acetivibrio cellulolyticus* [101], *Bacillus subtilis* [102], *Lactobacillus* [103], and *Staphylococcus* [104], have been effectively used for biotechnological applications. Depending on the biochemical or physical characteristics of foreign proteins, manifold bacterial hosts have been developed for surface display. However, it is difficult to achieve high-throughput screening of positive clones from libraries using a flow cytometer or microscope and to determine the levels of eukaryotic proteins using bacterial surface display technology.

### 6.2. Yeast Display System

The yeast *Saccharomyces cerevisiae* is well known as a useful host of genetic biotechnology because it can fold and glycosylate heterologous eukaryotic proteins. Furthermore, these cells are economically advantageous for high-density cultivation. Moreover, yeast cells can be used to express different proteins using several genetic markers. Indeed, various studies have reported that yeast can display different kinds of protein, the so-called “co-display” [105,106,107]. This molecular display system enabled us to perform high-throughput screening using conventional devices such as a flow cytometer or a multiwell plate reader [108,109].

The cell surface of the yeast *Saccharomyces cerevisiae* comprises β-glucans and mannoproteins [110], which exist outside the cell membrane. Cell wall proteins, such as agglutinins (Aga1 and Aga2), Flo1, Sed1, and Cwp1, are well-known anchor molecules that can retain target proteins on the yeast cell surface. In addition to these proteins, α-agglutinin is also one of the most widely used anchoring proteins for heterologous proteins in the yeast display system. A target and α-agglutinin fusion protein is produced by introducing multicopy plasmids or integrative plasmids into the host strain. A fusion protein in the system is transiently transported to the exterior of the cell membrane by secretory vesicles and then released by an enzymatic reaction involving phosphatidylinositol-specific phospholipase C. Finally, the target–α-agglutinin fusion protein is transferred to the cell wall [111,112]. Using the *Saccharomyces cerevisiae*–α-agglutinin display system, enzymes, fluorescent proteins [108], antibodies, and peptides [91] have been successfully displayed on the cell surface and used as elements of biomonitoring [113], adsorbents for screening of protein libraries [114], oral vaccines [115,116], catalysts in bioconversion, etc. Enzymes displayed on yeast cells can be repeatedly used via centrifugation with a synergistic conversion associated with the yeast intracellular metabolic pathway. Cells with the ability to degrade macroalgae and ferment macroalgal components have been developed by using molecular display technology, as described below.

### 6.3. Bioethanol Production from Laminarin

As described earlier, brown algae have the potential to be used to produce biomass energy because they do not compete with food and do not contain persistent lignin. As mentioned in the Introduction, brown algae contain up to 35% lignin on a dry weight basis [16] and have attracted much attention in the field of energy production. Nevertheless, they have not yet been effectively used as biomass because they cannot decompose into glucose. Therefore, to use brown algae, it is necessary to degrade laminarin to produce glucose for assimilation during alcohol fermentation.

Laminarinase, i.e., β-1,3-glucanase and β-1,6-glucanase, can produce glucose from polysaccharides for ethanol production [117]. Studies have reported ethanol production from laminarin using microorganisms [118,119,120]. *Pichia angophorae* can directly produce ethanol from laminarin [120]; however, it does not exhibit salt tolerance, unlike *Saccharomyces cerevisiae* [121]. In bioethanol production from brown macroalgae, the salt-tolerant characteristics of microbial cells would be advantageous. *Saccharomyces cerevisiae* has been used in the fermentation industry and can produce high levels of ethanol; however, it cannot assimilate laminarin.

Using qualitative proteomic analysis, Motone et al. reported that the marine, laminarin-assimilating bacterium *Saccharophagus degradans* strain 2–40 exhibits a high ability to degrade polysaccharides for bioethanol production [122]. In the multicomponent enzyme system of *S. degradans* 2–40, various polysaccharides, including agar, alginate, cellulose, hemicellulose, and laminarin, can be degraded [123]. In a proteomic study, 92 molecules, including 6 carbohydrases, were identified as proteins specifically produced during cultivation in a laminarin-containing medium [122]. Among the identified carbohydrases, Gly16G, Lam16B, and Gly5M belong to the glycoside hydrolase family 5 or 16 [122].

Although Gly16G and Lam16B have already been predicted to be laminarinases [123], the catalytic machinery of Gly16G is missing according to NCBI (http://www.ncbi.nlm.nih.gov/ accessed on 1 March 2023). Moreover, the molecular weight of Lam16B is extremely high and is therefore thought to be unsuitable for cell surface displays [124,125]. As a result, Gly5M was selected as the candidate hydrolytic enzyme for laminarin and displayed on the yeast cell surface. In the reaction between laminarin and Gly5M-displying yeast, oligosaccharides were produced, and Gly5M was suggested to be a novel hydrolytic enzyme for laminarin. Analysis of the produced oligosaccharides revealed that most comprised gentiobiose, with two glucose molecules linked by a β-1,6-glycosidic bond. Furthermore, *Aspergillus aculaeatus* β-glucosidase (BG)-displaying yeast was used to achieve ethanol production from laminarin. Cocultivation of Gly5M- and BG-displaying yeast strains was performed in a medium containing 20 g/L laminarin as the sole carbon source. As a result, 5.2 g/L of ethanol (corresponding to 46% of the theoretical yield) was produced under optimized conditions [122]. A study revealed that ethanol productivity depends on the initial inoculation ratio of the two yeast strains, and the proportion of the two enzymes is important in fermentation [122]. This cocultivation system using Gly5M- and BG-displaying yeast strains could be a powerful tool for ethanol production using laminarin in brown macroalgae.

### 6.4. Bioethanol Production from Xylan

Xylan is present in macroalgae and comprises a heteropolysaccharide with β-1,4-linked xylopyranoside. It constitutes >90% of the hemicellulose content [126]. Bioconversion of xylan into bioethanol can be an efficient and sustainable method for bioethanol production from nonedible biomass derived from macroalgae. In a previous study, xylan-degrading xylanase II (XYNII) from *Trichoderma reesei* and beta-xylosidase (XylA) from *Aspergillus oryzae* were codisplayed [127]. The XYNII- and XylA-displaying strain was used for direct ethanol production from birchwood xylan. The strain could produce D-xylose using the displayed enzymes, and fermentation of D-xylose was achieved by introducing the oxidoreductase-based enzymes NAD(P)H-dependent D-xylose reductase and xylitol dehydrogenase [128]. However, these enzymes cause intracellular redox imbalance and accumulation of by-products. An increase in cytosolic xylitol and glycerol leads to a decrease in the yield of ethanol [129,130].

Another route for the production of xylose involving isomerase (XI), which is predominantly derived from bacteria and catalyzes the isomerization of D-xylose into D-xylulose, has been investigated [131]. XI does not require coenzymes for isomerization. Moreover, using XI, higher theoretical yields (0.51 g ethanol/g xylose) can be achieved compared with the conventional pathway (0.46 g ethanol/g xylose) [132]. Ota et al. developed *Saccharomyces cerevisiae* displaying xylose isomerase (XylC) from *Clostridium cellulovorans* that can simultaneously isomerize and ferment D-xylose [133]. D-xylose isomerization in the cultivation medium has the potential to utilize D-xylose because D-xylulose is promptly absorbed by yeast cells via different uptake routes, unlike D-xylose uptake [134,135]. Nevertheless, ethanol production was low in this study [133] because of the limited catalytic activity of XylC. 

**Figure 2:**
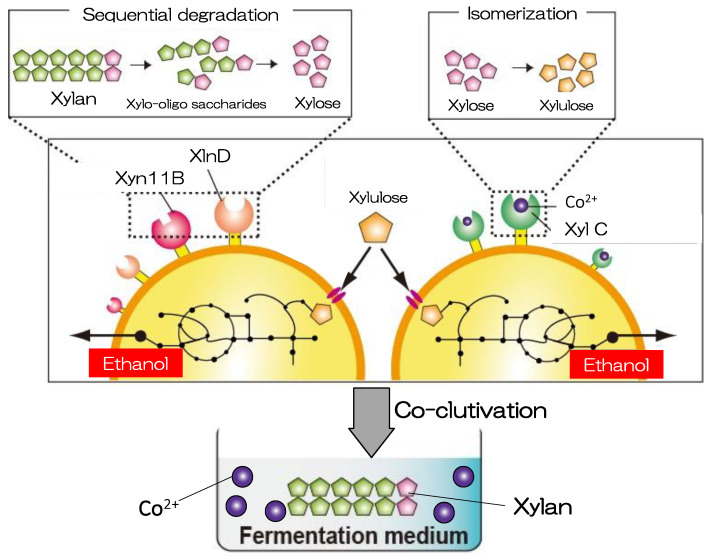
Coculture system designed for xylan saccharification and ethanol fermentation. The system is based on both xylanase-displaying strains and xylose isomerase-displaying strains. Xylan is degraded to D-xylose by the Xyn11B- and XlnD-codisplaying strain. This figure has been adapted from a previous study [137].

To enhance the catalytic activity of enzymes, it is important to optimize the concentration of specific cofactors because metal cations such as Co^2+^, Mg^2+^, or Mn^2+^ increase the activity and stability of XIs [136]. To improve the cell surface enzymatic activity of XylC-displaying yeast, specific metal cations have also been studied [137]. In this study, XylC-displaying yeast cells were cultivated and incubated in buffered solutions containing D-xylose and the following metal ions: Mn^2+^, Fe^2+^, Fe^3+^, Co^2+^, Co^3+^, Ni^2+^, Cu^2+^, and Mg^2+^. Co^2+^ markedly improved the catalytic activity of XylC on the yeast cell surface by 46-fold. As a result, Co^2+^ supplementation was introduced in the coculture system using two yeast strains, i.e., xylan-degrading *Saccharomyces cerevisiae* strain, which codisplays an endo-1,4-β-xylanase from *Saccharophagus degradans* 2–40 [138] and a β-xylosidase from *Aspergillus niger* [139], and a *Saccharomyces cerevisiae* strain that displays XylC (Figure 2). Supplementation of 3 mM Co^2+^ was the most effective cofactor for ethanol fermentation. In addition, the ethanol production rate and consumption rate of D-xylose were 38 ± 7.1 mg ethanol·g-cell^−1^·h^−1^ and 150 ± 3.6 mg xylose·g-cell^−1^·h^−1^, respectively. They were prominently improved compared with fermentation without Co^2+^ supplementation (6.3 ± 0.79 mg ethanol xylose·g-cell^−1^·h^−1^ and 56 ± 2.7 mg xylose·g-cell^−1^·h^−1^, 6.0- and 2.7-fold, respectively) [137].

Various enzymes from different microbial species may be an effective solution for fermenting xylan derived from macroalgae into ethanol. For example, the degradation of macroalgal xylan by xylanases from microbes such as *Cryptococcus* and *Thermomyces* has been investigated [140].

### 6.5. Bioethanol Production from Alginate and Mannitol

Introduction of the DEH transporter and components of the DEH metabolic pathway (DehR, KdgK, and KdgpA) into *Saccharomyces cerevisiae* is required for DEH assimilation because *Saccharomyces cerevisiae* cannot assimilate DEH. Enquist-Newmam et al. constructed a *Saccharomyces cerevisiae* strain that can use both DEH and mannitol [141]. To screen for a DEH transporter, the *Saccharomyces cerevisiae* strain BAL2193 was constructed by genomically integrating genes for DEH assimilation (*dehR* from *Sphingomonas* sp. strain A1, *kdgK* from *Saccharophagus degradans*, and *kdgpA* from *Vibrio splendidus*). Codon-optimized *dehR* from *Vibrio harveyi*, *kdgK* from *Escherichia coli*, and *kdgpA* from *Vibrio splendidus* were selected for engineering *Saccharomyces cerevisiae* using an enzymatic assay of the cell lysate and ethanol productivity. The resulting strain produced 36 g/L ethanol from a 98 g/L sugar mixture (alginate and mannitol). The metabolically modified *Saccharomyces cerevisiae* could generate ethanol from DEH and mannitol; however, unmodified *Saccharomyces cerevisiae* lacked the ability to utilize alginate [141].

Takagi et al., focused on secreted alginate lyases (Alg7D and Alg18J) and lipobox-containing cell-surface-attached alginate lyases (Alg7A and Alg7K) in *Saccharophagus degradans* and displayed them on *Saccharomyces cerevisiae* W303-1A [142]. Alg7A-, Alg7D-, and Alg18J-displaying strains exhibited endolytic alginate lyase activity, whereas the Alg7K-displaying strain exhibited exolytic alginate lyase activity. In addition to investigating the substrate specificity of the displayed alginate lyases, they produced yeasts codisplaying endolytic and exolytic alginate lyases. The degradation efficiency of these codisplaying strains was significantly higher than that of single alginate lyase-displaying strains. The Alg7A- and Alg7K-codisplaying strain had the highest alginate-degrading activity, producing 1.98 g/L reducing sugars [142].

**Figure 3:**
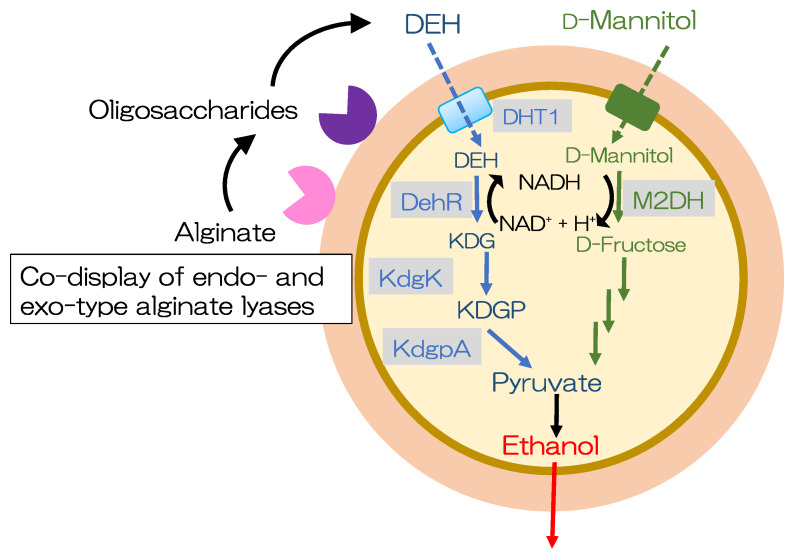
The cell surface display for direct bioethanol production from alginate and mannitol.

**Figure 4:**
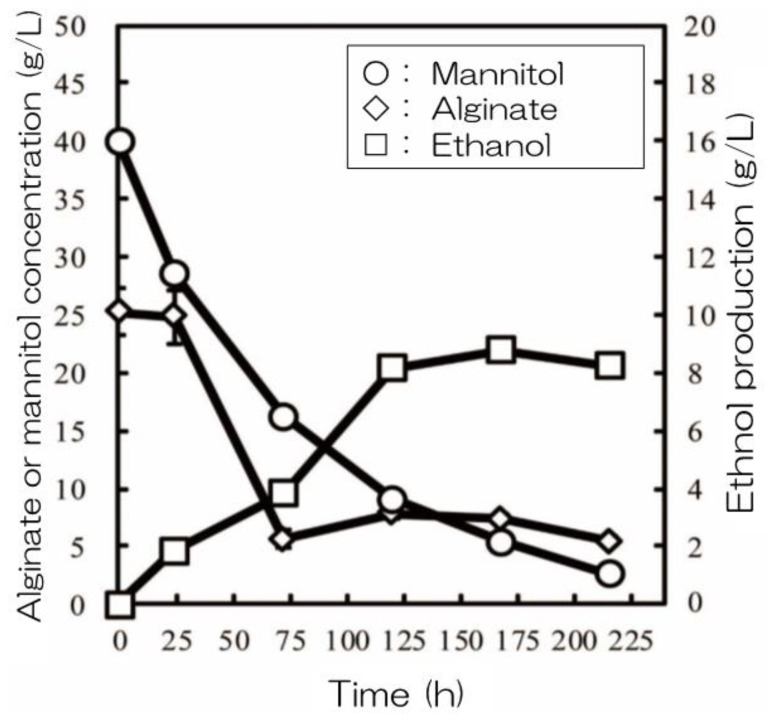
Direct ethanol production from alginate and mannitol using the alginate- and mannitol-assimilating strain. This figure has been adapted from a previous study [143].

Yeast molecular display technology has been further improved for direct ethanol production from alginate and mannitol in brown macroalgae (Figure 3) [143]. First, the genes encoding the components of the DEH pathway that produce ethanol directly from alginate and mannitol were examined. Then, the genes encoding *Alg7A* and *Alg7K* from *Saccharophagus degradans*, *DHT1* from *Asteromyces cruciatus*, *dehR* from *Vibrio splendidus*, and *kdgK* from *Escherichia coli* were examined. Furthermore, mannitol-metabolizing capacity was enhanced to control the redox balance during prolonged cultivation using a medium with mannitol as the sole carbon source. The resulting strain, alginate- and mannitol-assimilating (AM1), was cultivated in a medium containing 6% (*w*/*v*) of total sugar (approximately 1:2 ratio of alginate/mannitol). The strain could directly produce ethanol from alginate and mannitol and obtained 8.8 g/L of ethanol (Figure 4), with yields of up to 32% of the theoretical yield [143].

Alginate is initially degraded into oligosaccharides by Alg7A. These oligosaccharides are then sequentially degraded into monosaccharides by Alg7K. DEH is transported into the cytoplasmic space by the DEH transporter. Mannitol is converted to D-fructose by mannitol-2-dehydrogenase. This figure has been adapted from a previous study [143].

As another platform for bioethanol production using macroalgae, a cocultivation system using two different *Saccharomyces cerevisiae* strains, i.e., the cellulase-displaying strain (CDY) and AM1 strain, has recently been developed. The yeast CDY strain for ethanol production from glucan codisplays the cellulases endoglucanase, cellobiohydrolase, and BG on its cell surface [144].

Sasaki et al., developed a system using the *Saccharomyces cerevisiae* strains CDY and AM1 [145]. In the study, the acid hydrolysate of the brown macroalga *Ecklonia kurome* was used as the carbon source in the fermentation medium. The inoculation rate of cocultivated yeast is important for simultaneous utilization because there are varying amounts of carbohydrate components in brown algae. The cocultivation rate of the AM1 and CDY strains (4:1) was 2.10 ± 0.70 g/L of ethanol production. This yield was slightly higher than that produced by a monoculture of the AM1 strain and 2.1-fold higher than that produced by a monoculture of the CDY strain. In addition, peaks in mannitol, laminarin, and alginate stores in brown macroalgae appear around July, September, and from January to March, respectively [146,147]. Therefore, the harvesting season is also an important factor that should be examined to improve the production efficiency of ethanol and genetic and metabolic engineering.

### 6.6. Recovery of Metal Ions

For a long time, various types of bacteria have been examined in studies on the recovery of metal ions from aquatic environments [148,149,150,151]. Recently, algae have been shown to exhibit absorption abilities [151]. At present, macroalgae have better performance ability than microalgae, followed by cyanobacteria. For example, a study suggested that brown macroalgae have the most potential as bioadsorbents, with *Undaria pinnatifida* having an absorption ability of 0.6 mmol g^−1^ of total metals [152]. Another brown macroalga, *Fucus vesiculosus*, exhibited high absorption ability for Hg, Pb, and Cd [153]. Biopolymers derived from the alginate of *Ecklonia* sp. were suggested to be chelating materials for Pb^2+^, Cu^2+^, Cd^2+^, As^3+^, and Ag^+^, which are considered environmental pollutants [154]. Absorption using macroalgae for toxic metals and rare earth elements such as La, Ce, Pr, Nd, Eu, Gd, Tb, and Y, has also been investigated [155,156,157,158].

In a previous study on molecular display technology, metal-binding properties were endowed on yeast cell surfaces, and the engineered yeast cells were regarded as bioadsorbents [159,160]. These bioadorbents were used to recover metal ions from the extracts of macroalgae that absorb and enrich metal ions in the sea. The construction of yeast cells with the ability to absorb heavy metal ions, metal-binding proteins, and peptides has been demonstrated on the *Saccharomyces cerevisiae* cell surface. First, a hexa-histidine peptide (hexa-His) [161] and the metal-binding protein yeast metallothionein (YMT) [162] were displayed. The ability of the hexa-his- or YMT-displaying yeast strains to absorb Cu^2+^, Ni^2+^, or Cd*^2+^* was distinctively observed and enhanced compared with those of the control host strain. In addition, these yeast strains grew even in media containing Cu^2+^ or Cd^2+^ at toxic concentrations [161,162]. The separation process of cell-surface-bound heavy metal ions from the yeast cell surface was also developed for practical application in the bioremediation of contaminated hydrospheres. Furthermore, to enhance the utility of hexa-his- or YMT-displaying yeast strains for the recovery of metal ions, a system of self-aggregation of cells that bind to metal ions was introduced [163]. Because the Gts1 protein can induce strong aggregation when overexpressed, *GTS1* was expressed under the control of the *CUP1* promoter, which functions by increasing Cu^2+^ in the cells. Gts1-controlling aggregation of the resulting yeast strain was successfully achieved in response to Cu^2+^ in the medium. As another improvement in the metal-binding ability of *Saccharomyces cerevisiae*, multiple YMTs were displayed tandemly; this strain had an enhanced ability to recover metal ions depending on the frequency of tandem repeats [164]. The metal-binding ability of *Saccharomyces cerevisiae* endowed by molecular display systems has been applied not only to heavy metal ions but also to rare metal ions. For example, the ModE protein derived from *Escherichia coli* was displayed on the yeast, and its molybdate-binding ability was observed [165]. In addition, uranyl ions were recovered using yeast strains displaying NikR mutant proteins from *Escherichia coli* [166]. Further development of these metal-binding yeast strains could help in the recovery of various metal ions from extracts of macroalgae and other types of macroalgae.

## 7. Summary

We provided an overview of the bioconversion of macroalgae, mainly brown macroalgae, using microbial biotechnology. Terrestrial natural resources have met the expectations for food supply, energy, or valuable substances worldwide. To achieve sustainable development, biotechnological processes in particular have been developed and introduced into various processes, including the manufacturing and energy industries. To promote sustainability in these processes, it is not only important to use highly sophisticated technology but also to select natural resources. Therefore, the use of macroalgae instead of conventional terrestrial resources is considered suitable for these situations. Nevertheless, further screening of microorganisms from different regions is warranted to convert macroalgae into desirable feedstocks. In the future, genetic engineering, such as molecular display technology, will continue to provide ecofriendly tools for energy production and will be widely developed for the use of macroalgae.

Macroalgae contain several compounds for which there is high demand. A solution or technology that can be employed to preserve their habitat and marine resources should be discovered or developed to continue the harmonious use of macroalgae with the circle of life [167].

## Figures and Tables

**Table 1:** IC_50_ values of crude phlorotannins against fluorescent AGEs formation [66].

Algae (Original Area)	HAS-MGO (mg/mL)	BSA-MGO (mg/mL)
*Eck. Cava* (Mie)	0.53	0.51
*Eck. Kurome* (Fukuoka)	0.45	0.46
*Eck. Kurome* (Kumamoto)	0.53	0.50
*Cultured Eck. Kurome* (Kumamoto)	0.46	0.46
*Eck. Stolonifera* (Yamaguchi)	0.52	0.47
*Eis. arborea* (Mie)	0.46	0.53
*Eis. bicyclis* (Fukuoka)	0.45	0.43

The values of aminoguanidine hydrochloride (positive controls) were 0.70 mg/mL in the HSA-MGO model and 0.90 mg/mL in the BSA-MGO model.

**Table 2:** IC_50_ values of crude phlorotannins against fluorescent AGEs formation [71].

Algae (Original Area)	HAS-GA (mg/mL)	BSA-GA (mg/mL)
*Eck. Cava* (Mie)	0.70	0.75
*Eck. Kurome* (Fukuoka)	0.58	0.55
*Eck. Kurome* (Kumamoto)	0.61	0.59
*Cultured Eck. Kurome* (Kumamoto)	0.52	0.58
*Eck. Stolonifera* (Yamaguchi)	0.54	0.56
*Eis. arborea* (Mie)	0.51	0.61
*Eis. bicyclis* (Fukuoka)	0.48	0.52

The values of aminoguanidine hydrochloride (positive controls) were 1.10 mg/mL in the HSA-GA model and 1.93 mg/mL in the BSA-GA model.

## Data Availability

Not applicable.
